# A Framework for Estimating Economic Impacts of Ecological Restoration

**DOI:** 10.1007/s00267-024-02040-x

**Published:** 2024-09-26

**Authors:** Catherine Cullinane Thomas, Christopher Huber, Kristin E. Skrabis, Timothy B. Hoelzle

**Affiliations:** 1 U.S. Geological Survey, Fort Collins Science Center, Fort Collins, CO USA; 2 U.S. Geological Survey, Science and Decisions Center, Reston, VA USA; 3 U.S. Department of the Interior, Office of Policy Analysis, Washington, DC USA; 4 U.S. Department of the Interior, Office of Restoration and Damage Assessment, Washington, DC USA; 5 Present Address: U.S. Department of Agriculture, Economic Research Service, Washington, DC USA

**Keywords:** Ecological restoration, Economic impacts, Restoration economy, Restoration costs

## Abstract

Ecological restoration projects are designed to improve natural and cultural resources. Spending on restoration also stimulates economic impacts to the restoration economy through the creation or support of jobs and business activity. This paper presents accessible methods for quantifying the economic impacts supported by restoration spending and is written to be a guide and toolbox for an interdisciplinary audience of restoration practitioners and economists. Measuring the economic impacts of restoration can be challenging due to lacking or limited data. The complex, collaborative, and heterogeneous nature of restoration projects can make it difficult to clearly track costs, contributing to limited availability and inconsistency in restoration cost data. And business classification systems, such as the North American Industrial Classification System (NAICS), do not include restoration-sectors that consistently describe the patterns of restoration spending. The aims of this paper are to (1) provide restoration practitioners and program managers with a clear understanding of the application of economic impact analyses to restoration, (2) provide a framework for collecting project cost data for economic impact analyses, and (3) provide modeling best practices and an example application of the framework.

## Introduction

Spending on ecosystem restoration improves the functioning, resilience, and adaptability of ecosystems while supporting a substantial and growing restoration economy. Globally, the United Nation’s Decade on Ecosystem Restoration is building a world-wide movement to increase restoration efforts (United Nations 2021). Within the United States, spending on restoration has increased in recent years. A high-level accounting of the U.S. ecological restoration sector estimates that the restoration industry produced $10 billion in direct economic output (sales) in 2015; including ripple effects, ecological restoration activities supported an estimated total economic output of $25 billion and 221,000 jobs in the U.S. economy in 2015 (BenDor et al. 2015a). A 2023 follow-up study of the U.S. wetland and stream mitigation market found that this subset of the restoration economy grew at a compound annual rate of over 5% (BenDor et al. 2023).

The U.S. restoration economy is likely to continue growing in response to recent, substantial investments made by the U.S. Federal Government. The 2021 Infrastructure Investment and Jobs Act (also referred to as the Bipartisan Infrastructure Law [or BIL]), Executive Order 14008 “Tackling the Climate Crisis at Home and Abroad,” and the America the Beautiful Challenge explicitly appropriate a combined $3 billion in funds for remediation, mitigation, restoration, and conservation efforts on U.S. public and private lands (United States, Congress 2021; United States, Executive Office of the President 2021; United States, Executive Office of the President 2022a).^1^

Estimating the economic impacts of ecological restoration is important for ensuring that policy makers and the public can see the connections between the economy and policies and initiatives that support restoration. Economic impact studies can draw a clear line between jobs and business activity generated and supported by restoration funding. For example, a DOI study quantifying contributions to the national economy from the BIL funding highlights jobs supported by abandoned mine land reclamation, water resources investments, and orphaned well remediation and restoration (Braybrooks 2023). There have been similar studies estimating Federal program-level impacts; examples include the economic impacts of the National Oceanic and Atmospheric Administration’s coastal restoration program (Edwards et al. 2013), the U.S. Fish and Wildlife Service’s Partners for Fish and Wildlife Program and Coastal Program (Laughland et al. 2014), and the National Park Service’s restoration program (Cullinane Thomas et al. 2019). Like national analyses, state-level analyses can highlight jobs created or retained through state restoration programs; for example, both Massachusetts and Oregon have made connections between state forest and watershed restoration programs and local workforce development (Massachusetts Department of Fish and Game 2012; Nielsen-Pincus and Moseley 2013).

In addition to highlighting overall economic activity, state- and regional-level studies can also be used to inform and guide state and rural economic development efforts by pointing toward opportunities for development (BenDor et al. 2023). In some cases, local workforce development is an explicit goal of policies. One example is the U.S. Forest Service’s Collaborative Forest Landscape Restoration Program (CFLRP), which was established by Congress in 2009 to enhance forest and watershed health, reduce wildfire risk, and benefit rural economies. To measure the benefits to rural economies, the CFLRP requires project proposals and annual reports to include economic impact estimates (Hjerpe et al. 2021). Economic impact estimates can also be required (or used to support) policy analysis and land management planning. For example, analyses to determine how proposed projects will affect local economies and businesses are often conducted under the U.S. National Environmental Policy Act (NEPA), which requires U.S. Federal agencies to assess the potential environmental and community effects of proposed actions (Loomis 2002).

The aim of this paper is to provide restoration practitioners with a clear understanding of economic impact analyses and tools needed to estimate impacts for restoration projects and programs. The framework we present can be used to collect (or estimate) cost data and economic impacts for a wide range of restoration initiatives: large and small projects; past, present, future, or ongoing projects; and projects focused on mitigation, reclamation, remediation, or restoration. Jobs in restoration flow from the funds and resources used to plan, implement, and monitor projects. Therefore, to understand the economic impacts of restoration projects we must know how much money is spent on restoration and how those funds are distributed across economic sectors. For this reason, a main contribution of the paper is to provide a framework for collecting project cost data for economic impact analysis. We also describe methods for linking common restoration actions with standard economic sectors, using the North American Industrial Classification System (NAICS) as our example. Linking expenditures to standard economic sectors like NAICS bridges cost data to economic multiplier data used to estimate impacts. We demonstrate the framework using an example application designed to highlight and elucidate each element of the framework.

## Theoretical Background

**Fig. 1:**
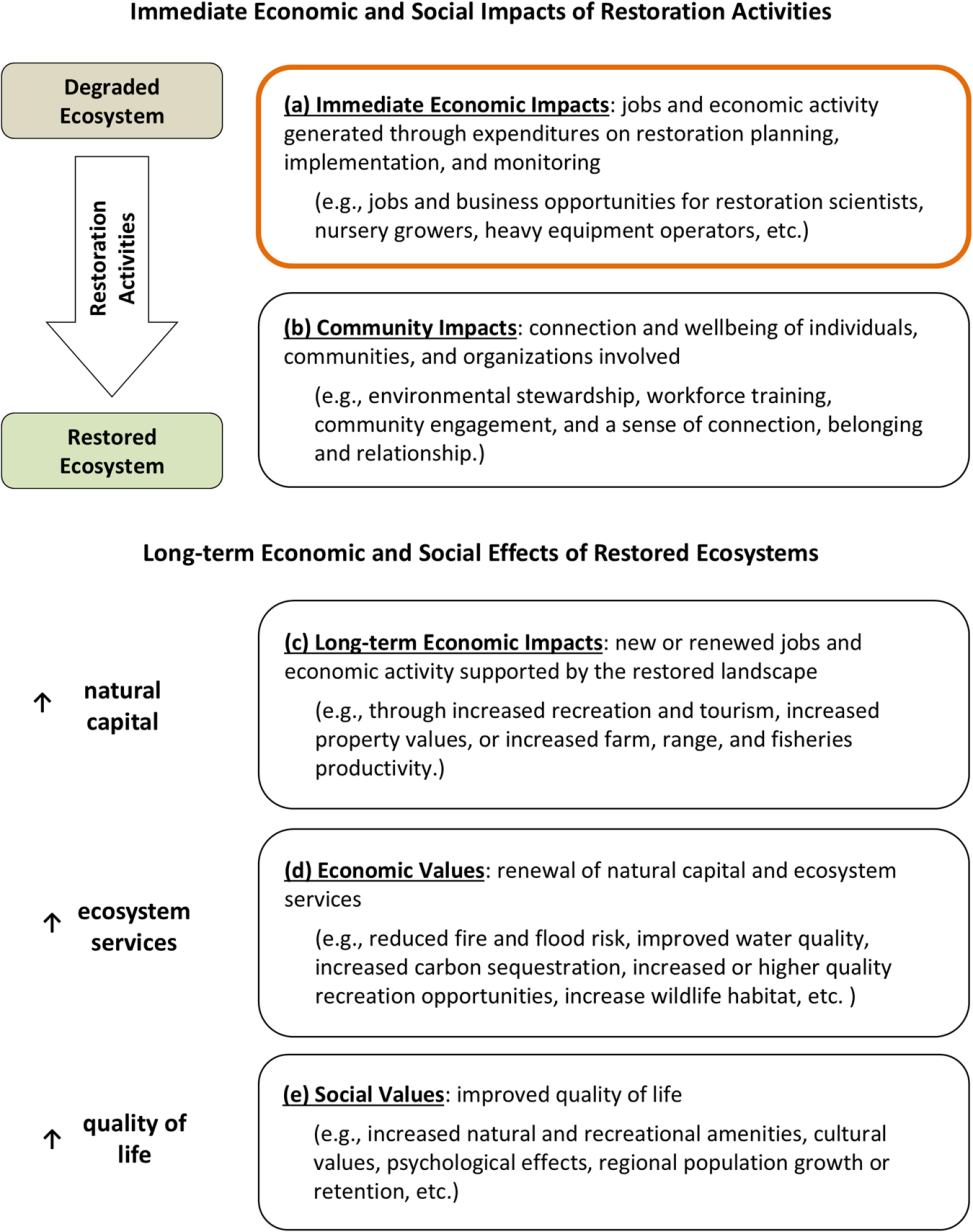
The potential socioeconomic benefits of restoration activities and restored ecosystems (Aronson et al. 2006; Baker 2005; BenDor et al. 2015a; Bodin et al. 2022; Liesch and Graziano 2023; Wortley et al. 2013). The immediate economic *impacts* of restoration (shown in box a) are the focus of this paper

Successful restoration projects transition degraded, damaged, or destroyed ecosystems into recovered states through restoration activities, and restoration activities and the resultant restored ecosystems can induce a chain of socioeconomic effects (Fig. 1). The socioeconomic outcomes of restoration unfold with time. Spending on restoration planning, implementation, and short-term monitoring stimulate immediate economic impacts to the restoration economy through the creation or support of jobs and business activity (Fig. 1a). Post-implementation, it takes time for a damaged ecosystem to recover to a “self-sustaining,” resilient system. This recovery period requires continued monitoring and support, including funding and human attention, which can further support restoration workers and businesses (Fig. 1a). Implementation and monitoring activities can also produce social impacts and values for individuals, communities, and organizations involved in restoration, including cultivation of environmental stewardship, workforce training opportunities, community engagement, and a sense of connection, belonging and relationship (Baker 2005; Fig. 1b).

When successfully restored, ecosystems can increase natural capital stocks and ecosystem service provisioning, which can result in long-term creation or renewal of jobs and economic activity through increased recreation and tourism activity, increased property values, or increased farm, ranch, and fisheries productivity (Aronson et al. 2006; Bodin et al. 2022; Fig. 1c). The renewal, or partial renewal, of natural capital and ecosystem services through restoration can also result in increased economic values of ecosystem services, such as reduced fire and flood risk, improved water quality, increased carbon sequestration, increased or higher quality recreation opportunities, and expanded wildlife habitat (Aronson et al. 2006; Fig. 1d). These outcomes can lead to improved quality of life for nearby residents through increased access to natural and recreational amenities, positive psychological effects stemming from living in a healthier environment, and regional population growth or retention (Liesch and Graziano 2023; Wortley et al. 2013; Fig. 1e). Studies that measure the long-term socioeconomic values of restoration projects compare the change in social welfare resulting from the restored ecosystem with the pre-restoration baseline.

This paper describes methods for estimating the immediate economic impacts of restoration projects (the first element in the chain of socioeconomic effects; Fig. 1a). Economic impact analyses of restoration activities measure the jobs and business activity created or supported through expenditures on restoration projects – thereby quantifying the “restoration economy.”^2^ Economic effects are described in terms of employment supported by restoration expenditures (i.e., jobs, if quantified annually, or job-years if quantified over longer periods of time);^3^ labor income (the wages and salaries earned by supported workers and proprietors);^4^ value added (the contribution of a project to regional gross domestic product (GDP));^5^ and economic output (the total value of the production of goods and services supported by a project).^6^ Project spending flows directly to the people, businesses, and organizations who plan, manage, implement, and monitor restoration projects. The employment of these people (jobs) and business activity (wages, profits, and taxes) are the direct economic effects of restoration. Restoration spending also creates a ripple effect, supporting additional jobs and business activity within a regional economy. Ripple, or multiplier effects, happen when directly affected businesses purchase goods and services from other businesses in the region (indirect effects), and when employees of affected businesses spend their wages and salaries in the region (induced effects); together, the indirect and induced effects are the secondary (or ripple) effects of restoration spending. Secondary effects extend to supporting sectors and occupations beyond those directly involved in restoration.

Direct and secondary effects to a regional economy can be estimated using multipliers derived from economic input-output (I-O) models. I-O models use data from national accounts to quantify how direct spending flows through supply chains within a region to create indirect and induced effects. The availability and specification of national accounts is specific to individual nations. In the United States, input-output models rely on the Bureau of Economic Analysis (BEA) benchmark input-output (I-O) national accounts (Bureau of Economic Analysis 2011). BEA I-O accounts define industry spending patterns for industry aggregates based on the NAICS^7^ industry classifications (note that there are no restoration industries defined in the I-O accounts or NAICS codes). Several commercial input-output models are available in the United States, including the BEA’s Regional Input-Output Modeling System (RIMS II)^8^ and the commercial Economic Impact Analysis for Planning (IMPLAN) model.^9^^,^^10^ Additional information about I-O models is included in the Appendix.

Economic impacts are measured and reported for a specified regional economy. Regional economies can be defined as the immediate local area around a project site, such as a county or several counties, or as a larger region, a state, or the entire Nation. The size of the economic impacts of a project directly correlates with the size of the selected regional economy, with smaller impacts for smaller regions; this is because only economic activity that happens within the defined regional economy is counted. Smaller and more rural regions tend to have greater economic “leakage” (i.e., a greater portion of dollars leaving the local economy) because fewer project inputs can be sourced from within the local region. The appropriate region to choose depends on the context and intent of the analysis. For an analysis intended to show the economic impacts of a major national program like the Infrastructure Investment and Jobs Act (BIL), a national region would be appropriate (e.g., Braybrooks 2023). For site-specific case study analyses of projects funded by national programs, it is possible to estimate impacts to both the national economy and the local economy near the project site (e.g., Cullinane Thomas et al. 2019). State programs may choose a state-level context to describe overall impacts to the state economy (e.g., Massachusetts Department of Fish and Game 2012), or may choose smaller sub-state regions to gain a better understanding of how restoration projects impact specific regions, especially rural or targeted regions within the state (e.g., White et al. 2016). Analyses focused on understanding the effects of restoration projects or programs on local employment and business opportunities will need to use smaller local areas that encompass the local economy of concern; this is relevant for policy and management analyses, like for NEPA planning, and for analyses of programs that have a specific objective of supporting local and rural economies. Capture rates (the portion of direct spending that stays within the local economy) are often small for rural areas; economic impact analyses of these regions can help identify strategic economic development activities that could increase local restoration business and employment opportunities (e.g., Hjerpe et al. 2021).

## Methodology

### Motivation

A primary challenge to estimating the economic impacts of ecosystem restoration is limited availability and inconsistency in restoration project cost data. The restoration economy is composed of a complex mix of funding sources and industries. Funding for restoration is driven by combinations of regulations and policies that mandate or incentivize restoration, Federal and State grants, State-level initiatives and legislation, and private investments by foundations, institutions, non-profits, and corporations (BenDor et al. 2015b). Project funding, leadership, resources, and labor are often pooled and pieced together across multiple public and private sources (Nielson-Pincus and Moseley 2010; Nielson-Pincus and Moseley 2013; Cullinane Thomas et al. 2016). The complexity of these collaborative efforts can make it difficult to clearly track costs, contributing to limited availability and inconsistency in restoration project cost data (Holl and Howarth 2000; Nielsen-Pincus and Moseley 2010; Robbins and Daniels 2012; BenDor et al. 2015a).

A further challenge to estimating economic impacts is that there are no restoration sectors defined within the current economic industrial classification scheme. Industries and trades required for restoration can include project managers, planners and consultants, scientists and engineers, landscape and nursery products providers, retail and whole-sale trades, heavy equipment owners and operators, and labor-intensive workers, all of whom are employed from a variety of businesses, non-profit organizations, universities, and governmental institutions (Nielson-Pincus and Moseley 2010; Cullinane Thomas et al. 2016). Having no defined restoration sectors makes estimating restoration impacts more difficult than for traditional economic sectors.

### Development

Because there are no restoration sectors in the current NAICS scheme, spending patterns for restoration activities are not defined in the BEA I-O data. There have been two main approaches for dealing with this: (1) break restoration activities into parts that can be reasonably represented by existing NAICS codes, or (2) create custom spending patterns for restoration activities. Approach (1) is the most common approach (see, for example, Massachusetts Department of Fish and Game 2012; Laughland et al. 2014; BenDor et al. 2015a; Liesch and Graziano 2023; Botta et al. 2022; Braybrooks 2023; BenDor et al. 2023).

Alternatively, Nielsen-Pincus and Moseley, in their 2010 study of the economic contributions of watershed and forestry restoration projects in Oregon, pioneered an approach to create custom spending patterns for restoration (Nielsen-Pincus and Moseley 2010; Nielsen‐Pincus and Moseley 2013). To do this, they surveyed contracted businesses and asked them to report typical expenditure patterns, including information about portions of spending made locally. They combined these survey data with program grant data to develop custom production functions for different restoration contractor types (labor intensive contractors, equipment intensive contractors, and technical planning and design contractors) and different forest and watershed restoration project types (in-stream, riparian, wetland, fish passage, and upland).

The methods developed in Nielsen-Pincus and Moseley (2010) were further developed and modified in White et al. (2015) and (2016) to estimate impacts of U.S. Forest Service restoration strategies in Oregon, and in Cullinane Thomas et al. (2016) to develop a series of case studies to estimate economic contributions of a variety of restoration projects associated with U.S. Department of the Interior lands and programs. Each of these studies used primary survey data to create detailed custom spending patterns for restoration activities, providing region and context-specific information that is not available in the “out-of-the-box” sectors in models like RIMS II and IMPLAN. These custom data collections can provide clearer pictures of the economic impacts of restoration activities, especially for smaller and more rural regions.

However, there are tradeoffs to this approach. Collecting primary data is costly and time consuming, and project contractors can be reluctant or unwilling to complete business surveys, leading in some cases to high non-response rates (Cullinane Thomas et al. 2016). Furthermore, custom production functions can be difficult to generalize or transfer because data collected for one region and restoration type cannot be assumed to represent another region or restoration type (Cullinane Thomas et al. 2016; Hjerpe et al. 2021).

Here we present a restoration cost collection framework that represents a hybrid of approaches (1) and (2) and draws on experience gained through developing over 30 case studies of the economic impacts of restoration across a wide variety of ecosystems and project types (Department of the Interior 2012; Cullinane Thomas et al. 2016; Cullinane Thomas et al. 2019; Huber et al. 2019; Huber et al. 2020). The framework prescribes methods most like the studies described in approach (1), matching restoration activities to best-fit NAICS codes, but with the collection of additional data for activities and institutions that are not well-represented by existing NAICS codes. Specifically, more detailed data are collected for project management, planning, and monitoring activities undertaken by governments and non-governmental organizations (NGOs). Additional data are also collected to allow for estimates of local capture rates and to identify supply chain levels for materials purchases.

### The Restoration Cost Collection Framework

**Fig. 2:**
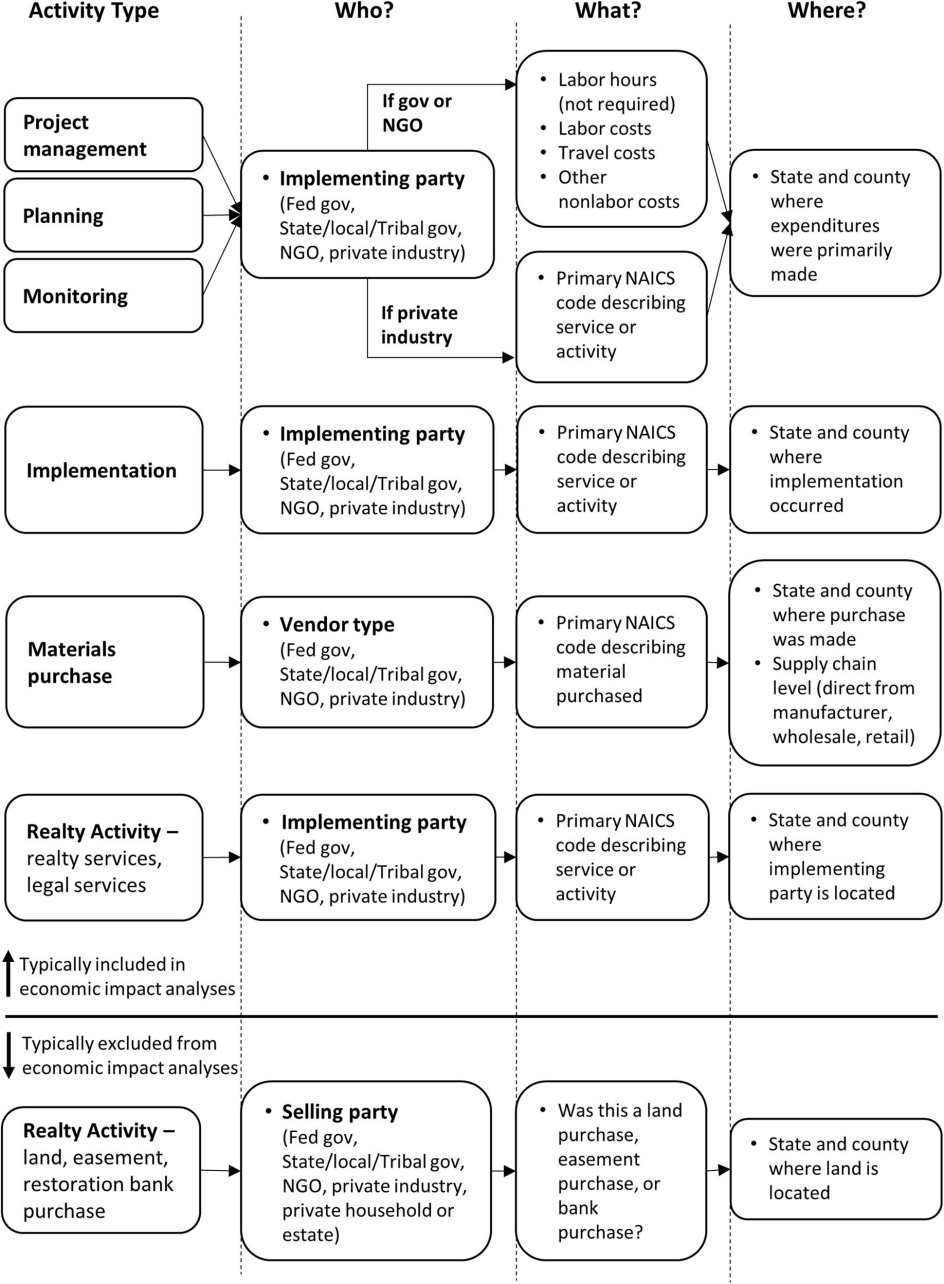
The Restoration Cost Collection (RCC) framework is organized around the information needed to estimate economic impacts, specifically: How much was spent? by whom? on what? when? and where? This flow chart shows RCC ”who, what, where?” questions for restoration expenditures based on restoration activity types. Restoration activities are defined as the components of a project for which costs can be assigned. Restoration activity types include project management, planning, monitoring, implementation, materials purchases, and realty activities. [NGO: Non-Governmental Organization; Fed gov: Federal government; NAICS: North American Industrial Classification System]

The Restoration Cost Collection (RCC) framework is organized around the information needed to estimate economic impacts, specifically: How much was spent? by whom? on what? when? and where? The system also captures data on the composition of projects. Projects are broken into *restoration activities*, which are broadly defined as components of a project for which costs can be assigned. Restoration activities are further classified into activity types: project management, planning, monitoring; implementation; materials purchases; and realty activities. Breaking the project up by activity type provides useful project composition data and enables collection of more nuanced “who, what, and where” responses (Fig. 2 describes the additional “who, what, where” data collected for each activity type). For most activities, spending is matched to a NAICS sector to describe the work or purchase made (Tables A1–A4 provide example NAICS codes for each of the restoration activity types).

The way that restoration activities are defined for a project is flexible and allows for varying levels of detail to be collected, depending on data availability. Project components can be combined into relatively few activities or divided into detailed line items. Activities can be detailed and include activities such as equipment rental, design work, surveys, propagation and rearing, seed collection, and bank stabilization. Alternatively, activities can be much broader, for example wetland creation, cropland conversion, and dam removal. The more refined and detailed the activity cost breakdown, the better the economic analysis. Cost and project data are also improved when separate line items are recorded whenever who, what, where, or when elements differ within an activity. For example, if two different contractors (who) located in different areas (where) are hired to do similar work, it is best to list these costs separately. Similarly, if an activity takes place over multiple years (when), it is best to keep separate annual line items for this activity.

#### Project management, planning, and monitoring activities

Project management, planning, and monitoring activities are often implemented by NGOs or by Federal, State, local, or Tribal government agencies; in some cases, activities are implemented by private management or consulting firms (Cullinane Thomas et al. 2016). Although NAICS sectors for governments exist, in many cases these sectors broadly describe the spending patterns of the whole of government. Similarly, NAICS sectors for NGOs typically describe all grantmaking and giving services. This makes matching government and NGO work to NAICS sectors problematic. For this reason, if the implementing party for project management, planning, or monitoring activities is a government or NGO, then information on labor, travel, and other nonlabor costs is collected (instead of a NAICS code). Project management, planning, and monitoring activities implemented by private industry are matched to the NAICS code that best describes the professional, scientific, or technical services provided (see Table A1 in the appendix for a list of relevant project management, planning, and monitoring NAICS codes).

#### Implementation activities

Implementation activities include the “on-the-ground” (i.e., physical implementation) work of a restoration project. This could include earth-moving, invasive species removal, fuels reduction, demolition, planting, construction, and on-going post-installation maintenance of the site. All implementation activities, regardless of the implementing party, are matched to NAICS codes (see Table A2 in the appendix for a list of relevant restoration implementation NAICS codes).

#### Materials purchases

The materials purchase activity type is reserved for materials that constitute a major element of project expenditures. For example, the materials purchase activity might be used for a culvert purchase for a stream restoration project, or a nursery product purchase for a planting project. Other, smaller materials purchases are often included within implementation expenditures and do not need to be listed as line items. All materials purchases, regardless of vendor type, are matched to NAICS codes (see Table A3 in the appendix for a list of relevant materials NAICS codes). Materials purchases include an extra question about the supply chain level of the purchase so that expenditures can be matched to the right type of supplier (Nielsen-Pincus and Moseley 2010). Specifically, the RCC collection asks: was the purchase sourced direct from the manufacturer/producer, from a wholesaler, or from a retail store?

#### Realty activities

Realty activities, such as land purchases, easement purchases, and restoration bank purchases, are common restoration activities and can constitute large portions of total project costs (Cullinane Thomas et al. 2016). These purchases can be problematic with respect to economic impact analyses—money and land change hands, but it is difficult to know if and how this transaction affects jobs and business activity without additional information on how landowners spend the money they receive for the transaction (Seidl et al. 2018). To illustrate, consider a landowner who sells their property or sells an easement on their property. This landowner will receive payment for the sale—they could use the funds to purchase other land, save all or a portion of the funds, or spend all or a portion of the funds on unknown goods and services. Without additional information, it is not possible to know if, how, or when these funds will be spent within the regional economy, and it is likely that much of the payment will be spent outside of the region or spent in future years. Therefore, funds for easements and land purchases are typically excluded from economic impact calculations unless additional information about how funds are spent is available.

Transaction costs for land purchases and easement servicing costs are, however, often known and can have important effects on local businesses. Therefore, the RCC framework lists realty transaction and management costs as separate line items in the cost collection system so that they can be matched to NAICS codes and included in impact analyses (see Table A4 in the appendix for a list of realty activity NAICS codes).

#### Example application

We demonstrate an economic impact analysis using the RCC framework for a hypothetical restoration project. The goal of this example is to represent a range of potential restoration activity types and scenarios, and to show how the collected data can be used to estimate the economic impacts of a project.

This example project was funded through a grant from the Commonwealth of Pennsylvania, with additional support from a local conservation NGO. Pennsylvania was interested in the economic impacts of the project to the state economy. The project site was in Wyoming County, Pennsylvania. Project goals were to improve aquatic and terrestrial habitat along the Susquehanna River while also improving outdoor recreation opportunities. Main project activities included dredging, recontouring, and revegetating the riparian area of a two-mile stream segment; a small dam removal; construction of a multi-use recreation trail; and purchase of adjacent land intended to help improve wildlife connectivity with the restored stream segment. Planning began in October 2016, and the overall project concluded in September 2019. Total spending on the project over the three-year period was $7,679,700 (nominal expenditures, i.e., not adjusted for inflation).

Appendix Table A5 shows project activity data collected for the full lifecycle of the project. Activities occurred at all phases of the project (planning, monitoring, and implementation) and were spread out over the three-year project timeline. Data include the activity name, activity type, cost, and date range for 25 restoration activities. The table further specifies the implementing party, the state and county (or county equivalent) where each firm, organization, or selling party was located, and the best-fit NAICS code for each activity (or the expenditure breakdown for NGO and government planning, management, and monitoring activities).

Project planning, management, and oversight activities were primarily conducted by the Commonwealth of Pennsylvania (Table A5, Activities 1 and 4), with project engineering and design and archaeological surveys contracted to a private engineering firm (Table A5, Activities 2 and 3). Monitoring activities were conducted before and after project implementation and included water quality monitoring and biologic surveys completed by State and private parties (Table A5, Activities 5–9). There were several large materials purchases that were separated into their own line items; these included rocks and riprap, grass and shrub seeds, and tree sapling purchases (Table A5, Activities 10–13).

Project implementation, including dam removal, installation of fish passage structures, bank stabilization, erosion control, recontouring, and instream dredging were completed by Pennsylvania-based private engineering and construction firms (Table A5, Activities 14–16). As part of implementation, a New York firm was hired to bury electrical lines (Table A5, Activity 17). Firms were also hired to construct a multi-use hard surface outdoor recreation trail and install an adjacent safety fence (Table  A5, Activities 18–19). Riparian planting was completed by a conservation NGO (Table A5, Activity 20), and educational interpretation signs were designed, fabricated, and installed by an environmental education NGO (Table A5, Activity 21). All construction activities and electrical utility work required hiring firms to complete inspection (Table A5, Activities 22–23).

A 10-acre plot of land was purchased from a private seller and was secured using legal services from a local attorney (Table A5, Activities 24–25). Following acquisition, the property was placed into a conservation easement to protect the land into perpetuity.

This hypothetical project demonstrates elements of the RCC framework. Notice that the planning, project management, and monitoring activities completed by the State government or by NGOs list expenditure breakdowns instead of NAICS codes (Table A5, Activities 1, 4, 5, 7, and 8); whereas all other activity types completed by these types of organizations use NAICS codes (Table A5, Activities 13, 20, and 21). Recall that cost and project data are improved when separate line items are recorded whenever who, what, where, or when elements differ within an activity. Several activities in the cost table demonstrate this principle. For example, fish monitoring surveys were completed in 2017 (prior to project implementation) and then again in 2019 (following implementation); although these activities were completed by the same State agency, costs are included as separate line items because they happened in different years (Table A5, Activities 7 and 8). Similarly, two different riprap purchases were made in separate years (Table A5, Activities 10 and 11). Also recall that funds for easements and land purchases are typically excluded from economic impact analyses (unless data on landowner expenditure patterns are available, Seidl et al. 2018), but transaction costs, such as realtor and legal fees, are typically included (Table A5, Activities 24 and 25).

We used the IMPLAN model and data system to estimate the economic impacts of our hypothetical Pennsylvania stream restoration project (IMPLAN model, 2019 Data). To estimate the economic impacts of this project, NAICS codes and expenditure breakdowns were bridged to economic sectors in the IMPLAN model. Details of how project expenditure data were modeled in the IMPLAN software is included in the Appendix (Table A6).

We chose the Commonwealth of Pennsylvania as the regional economy to highlight the economic impacts to the State (note that we could have chosen a smaller region, such as Wyoming County or a set of counties including Wyoming County, to understand more local effects of the project). Defining the regional economy as the Commonwealth of Pennsylvania means that only the economic activity that takes place within Pennsylvania is included in impact results. Project expenditures awarded to firms hired from outside Pennsylvania, as well as secondary economic activity from out-of-state firms, are “leaked” from the model and do not generate economic effects in the region. Of the total $7,679,700 in project spending, $6,709,200 (87%) was captured and included in the Pennsylvania State impact analysis (directly leaked expenditures include $220,500 awarded to contractors located outside of Pennsylvania and $750,000 spent on the conservation easement purchase).

Table 1 shows estimated economic impacts to the Commonwealth of Pennsylvania (with impact results inflated to 2019 dollars). The project directly supported an estimated 40 job-years, $2,700,000 in labor income, $3,867,000 in value added, and $7,009,000 in direct economic output within the State of Pennsylvania. These direct effects describe the jobs and economic activity realized by the primary businesses hired to do the restoration work. The project supported an additional 14 indirect job-years, $993,000 in indirect labor income, $1,571,000 in indirect value added, and $2,918,000 in indirect economic output. These indirect effects measure the supply chain effects from Pennsylvania businesses’ purchasing intermediate goods and services from each other. The project further supported 21 induced job-years, $1,161,000 in induced labor income, $1,956,000 in induced value added, and $3,321,000 in induced economic output. These induced effects measure the ripple effects of workers spending their wages on household goods and services within the State. Altogether, the project supported an estimated total of 75 job-years, $4,854,000 in labor income, $7,394,000 in value added, and $13,248,000 in economic output within the Pennsylvania State economy (Table 1).

**Table 1:** Economic impact results for example Pennsylvania stream restoration project

Effect	Job-years	Labor income ($)	Value added ($)	Economic output ($)
Direct	40	2,700,000	3,867,000	7,009,000
Indirect	14	993,000	1,571,000	2,918,000
Induced	21	1,161,000	1,956,000	3,321,000
Total	75	4,854,000	7,394,000	13,248,000

Results give the estimated direct, indirect, induced, and total effects measured in job-years, labor income, value added, and economic output (in 2019 dollars) supported over the life of the project (2016–2019) for businesses and organizations located within the state of Pennsylvania. Economic impacts were estimated using project expenditure data organized using the Restoration Cost Collection (RCC) framework (see Table A5) applied to the IMPLAN input-output model (IMPLAN model 2019 data; see Table A6)

## Discussion

Spending on ecosystem restoration is growing, spurred by initiatives such as the United Nations Decade on Ecosystem Restoration and the U.S. Infrastructure Investment and Jobs Act (United Nations 2021; United States Congress 2021). Quantifying the direct and ripple effects of ecosystem restoration to regional and national economies can help to justify continued support in building a robust restoration economy. This paper presents accessible methods for quantifying the jobs and business activity supported by restoration spending. The RCC framework presented here is applicable to a broad range of project and program-level analyses, including mitigation, reclamation, remediation, and restoration projects. The primary focus of the framework is methodology to collect the restoration cost data needed to describe how project spending flows through supply chains. With these data, it is possible to measure how regional economies are directly affected by restoration efforts; and, in cases where economic multipliers can be estimated, these data can be used to quantify the ripple effects of restoration throughout an economy.

A template of the RCC framework is included in the Supplementary materials (Supplement 1). When possible, we recommend keeping track of project expenditures as they occur. Small or related projects can be combined to describe the overall impacts of a program or a case (see Cullinane Thomas et al. 2019 for an example of combining small projects to describe the impacts of a restoration program). For projects that have already been completed, cost data can be collected by querying or reorganizing existing cost data (see Huber et al. 2020 for an example retrospective study that reorganizes existing cost data). For future or proposed projects, the RCC form can be filled with planned or projected cost estimates. Once restoration costs are compiled or estimated, economists and analysists experienced with economic input-output modeling can estimate the economic effects of projects to regional, state, or national economies.

To overcome data collection issues related to the complex and collaborative nature of restoration projects, restoration cost collection efforts can be centralized, with a primary project manager or coordinator designated with the responsibility of keeping the RCC data updated. Restoration projects can take many years, especially with sufficient monitoring for restoration success; therefore, it can be helpful to develop a strategy about how these data will be collected, maintained, and updated over the lifespan of the project. In some cases, it may be necessary to make cost data collection a mandatory reporting requirement.

The RCC framework can also be used to build a library of cost data, enabling consistent project expenditure data that can be easily compared across a range of project types and scopes. Collected data describe how project costs are distributed across planning, implementation, and monitoring, which can help programs and policymakers better understand the distribution of restoration costs across project phases. Following restoration implementation, practitioners typically identify the need for corrective actions using adaptive management and evaluate the success of the project through long-term monitoring. While monitoring and adaptive management costs may incur over many years or even decades, they can also be evaluated in the RCC framework to determine the full costs of restoration projects from inception through monitoring. Additionally, this information can be used to determine the effectiveness of the money spent toward achieving the desired ecological state.

Because there is not yet a formal restoration industry defined and tracked by the BEA, estimating the economic impacts of restoration projects requires either approximating industry spending patterns by matching elements of a project to existing industries or using primary survey data to construct custom spending patterns for the target project or project type. The RCC framework presented in this paper leans toward the simpler approach of using data and multipliers from existing industries. This is a common approach because the required data are relatively easy to obtain.

However, an important limitation to the RCC approach is that data from other industries may not be a good approximation of the economic activity of some restoration projects. This can be an especially important consideration for analyses that seek to examine and identify economic activity and development opportunities of regions with nascent or growing restoration economies, or for analyses of restoration programs that have niche industry linkages. For example, Hjerpe et al. (2021) emphasized the need for contractor surveys to understand wood utilization from forest stewardship contracts for a large-scale restoration program in Arizona. For this program, data were needed to understand the types and locations of mills used to process thinned timber. With these data, the authors were able to identify local industry gaps where business activity was leaked to mills outside of the region and recommend opportunities for regional economic development. Hjerpe et al. (2021) also found that, even with custom restoration spending patterns developed for similar restoration types, patterns developed for other (or broader) regions may poorly represent the target region. These results suggest that studies that require precise results will likely need primary studies and data collection.

Although the framework presented here is focused on North America, information about economic impacts of restoration can be important in countries with extensive natural resource degradation and/or developing economies. The economic concepts and cost collection system described in this paper are relevant to all places. However, economic input-output and multiplier data are less readily available for many countries. If multiplier data are not available, researchers may be able to develop multiplier estimates using regional statistical data and national systems of accounts (see Miller and Blair 2009 or Loomis 2002 for methods). Alternatively, researchers could rely on rough multiplier estimates from literature to approximate impacts (see Cullinane Thomas et al. 2016 for cautions about transferring multiplier estimates across studies).

Even in cases where economic multiplier data are unavailable (or prove too cumbersome to estimate given available data and resources), collecting these cost data may be useful for purposes of future project planning and budget formulation. And the collection process may help to illuminate gaps and opportunities for restoration-related businesses development in a region. Further, if national or regional information on industry jobs-per-output values and industry wage rates are available, it is possible to estimate direct effects. To do this, direct output is estimated as total restoration spending within the region. Direct jobs are then estimated as direct output multiplied by jobs-per-output; and direct labor income is estimated as direct jobs multiplied by industry wage rates.

Ecological restoration induces a chain of societal benefits, and these benefits are under-reported (Aronson et al. 2010). Estimating and reporting the economic impacts of ecological restoration can help policy makers and the public see how restoration funding supports regional and national economies, and can help planners, economic developers, and entrepreneurs identify growth opportunities in the restoration economy. A limitation of the RCC framework is that it is not practical for measuring the overall economic impacts of a Nation’s restoration economy; this would require national accounts to delineate and measure the restoration economy, much like is done for the outdoor recreation economy (Bureau of Economic Analysis 2023a), the marine economy (Bureau of Economic Analysis 2023b) and proposed broader environmental-economic accounts (Fixler et al. 2023). Without a broader delineation, it is difficult to measure the scale and growth of the overall restoration economy. Systematically integrating restoration industries into the system of national accounts (as is proposed in BenDor et al. 2023) would be a step toward tracking the size and growth of the restoration economy. In the meantime, restoration practitioners can help to tell this story by measuring and reporting the regional economic impacts of their projects and programs.

## Supplementary Information

Supplementary information

## Appendix

**Overview of Economic Input-Output Models**

Input-output (I-O) models are based on an accounting framework that describes the interacting business transactions that take place in a defined economy (typically a national or subnational economic region) over a defined period of time (typically one year) (Jackson 2020; Miller and Blair 2009). Consider an economy with *J* industries. An individual industry *i* can sell goods and services to other industries *1, …, j* for use as inputs (called intermediate demand) and can sell to final consumers such as households, governments, and exports (called final demand). The total output produced in an economy, ***X***, is the sum of all intermediate demand plus the sum of final demand (Eq. 1).

1 $$\left[\begin{array}{c}{x}_{1}\\ \vdots \\ {x}_{j}\end{array}\right]=\left[\begin{array}{ccc}{x}_{11} & \cdots & {x}_{1j}\\ \vdots & \ddots & \vdots \\ {x}_{j1} & \cdots & {x}_{{jj}}\end{array}\right]+\left[\begin{array}{c}{y}_{1}\\ \vdots \\ {y}_{j}\end{array}\right]$$

From Eq. 1, a *direct requirements matrix* ***A*** can be defined that describes the proportions of inputs from industries *1, …, j* that are required to produce the output of each industry *i* (Eq. 2).

2 $$\left[\begin{array}{c}{x}_{1}\\ \vdots \\ {x}_{j}\end{array}\right]=\left[\begin{array}{ccc}{a}_{11} & \cdots & {a}_{1j}\\ \vdots & \ddots & \vdots \\ {a}_{i1} & \cdots & {a}_{{jj}}\end{array}\right]\left[\begin{array}{c}{x}_{1}\\ \vdots \\ {x}_{j}\end{array}\right]+\left[\begin{array}{c}{y}_{1}\\ \vdots \\ {y}_{j}\end{array}\right]$$

***A*** describes the direct spending pattern (also called a production function) required to produce ***X***. Equation 2 can be written as ${\boldsymbol{X}}={\boldsymbol{AX}}+{\boldsymbol{Y}}$. Solving for ***X*** gives Eq. 3, where ***I*** is a JxJ identity matrix, and ${\left({\boldsymbol{I}}-{\boldsymbol{A}}\right)}^{{\boldsymbol{-}}{\boldsymbol{1}}}$ is called the *total requirements matrix*.

3 $${\boldsymbol{X}}={\left({\boldsymbol{I}}-{\boldsymbol{A}}\right)}^{-{\bf{1}}}{\boldsymbol{Y}}$$

The elements of the total requirements matrix describe the multiplier effect, giving the total output from all industries *1, …, j* required to deliver an additional dollar of final demand for industry *i*. The indirect multiplier effect, which stems from the ripple of backwards linked business-to-business transactions required to produce a final good, is equal to the difference between the total requirements matrix and the direct requirements matrix.

To estimate induced multiplier effects, which stem from households earning and spending wages in the regional economy, the I-O model needs to be closed with respect to households (Jackson 2020; Miller and Blair 2009). This means that Eq. 1 needs to be modified to exclude households from the final demand vector ***Y*** and instead include household wages and spending as a row and column in the transaction matrix ***X***. For a more detailed explanation of I-O models see, for example, Jackson (2020) or Miller and Blair (2009).

**Bridges between NAICS codes and common restoration activities**

Tables A1–A4

**Table A1:** North American Industrial Classification System (NAICS) codes relevant to restoration project management, planning, and monitoring activities. Codes are based on 2017 NAICS (Office of Management and Budget 2017; see https://www.census.gov/naics/)

NAICS code	NAICS description
54121	Accounting, Tax Preparation, Bookkeeping, and Payroll Services
54161	Management Consulting Services
54162	Environmental Consulting Services (includes ecological restoration consulting services and wetland restoration planning services)
54182	Public Relations Agencies
54194	Veterinary Services
54199	All Other Professional, Scientific, and Technical Services
5411	Legal Services
5413	Architectural, Engineering, and Related Services (includes landscape architecture, building inspections, surveying and mapping, and testing laboratories)
541511	Custom Computer Programming Services
541512	Computer Systems Design Services
54169	Other Scientific and Technical Consulting Services
5417	Scientific Research and Development Services
813	Religious, Grantmaking, Civic, Professional, and Similar Organizations

**Table A2:** North American Industrial Classification System (NAICS) codes relevant to restoration implementation activities. Codes are based on 2017 NAICS (Office of Management and Budget 2017; see https://www.census.gov/naics/)

NAICS code	NAICS description
11194	Hay Farming
11199	All Other Crop Farming (includes grass seed farming)
11251	Aquaculture
11291	Apiculture
11292	Horses and Other Equine Production
11311	Timber Tract Operations
11321	Forest Nurseries and Gathering of Forest Products
11331	Logging
11411	Fishing
11421	Hunting and Trapping
11511	Support Activities for Crop Production (includes aerial spraying, soil preparation, planting, cultivating)
11521	Support Activities for Animal Production (includes breeding services, boarding)
11531	Support Activities for Forestry (includes estimating timber, forest firefighting, forest pest control, aerial seeding)
21231	Stone Mining and Quarrying
21232	Sand, Gravel, Clay, and Ceramic and Refractory Minerals Mining and Quarrying
21311	Support Activities for Mining (includes drilling, taking core samples, making geological observations)
22131	Water Supply and Irrigation Systems (includes operation of water treatment plants and/or water supply systems)
22132	Sewage Treatment Facilities (includes operation of sewer systems or sewage treatment facilities)
33661	Ship and Boat Building and Repair
111421	Nursery and Tree Production
1121	Cattle Ranching and Farming
221111	Hydroelectric Power Generation
221112	Fossil Fuel Electric Power Generation
221113	Nuclear Electric Power Generation
221114	Solar Electric Power Generation
221115	Wind Electric Power Generation
221116	Geothermal Electric Power Generation
221117	Biomass Electric Power Generation
23622-New	Commercial and Institutional Building Construction (includes new work)
23622-Repair	Commercial and Institutional Building Construction (includes reconstruction, rehabilitation, and repairs)
23711-New	Water and Sewer Line and Related Structures Construction (includes new work)
23711-Repair	Water and Sewer Line and Related Structures Construction (includes reconstruction, rehabilitation, and repairs)
23713-New	Power and Communication Line and Related Structures Construction (includes new work)
23713-Repair	Power and Communication Line and Related Structures Construction (includes reconstruction, rehabilitation, and repairs)
23731-New	Highway, Street, and Bridge Construction (includes new work)
23731-Repair	Highway, Street, and Bridge Construction (includes reconstruction, rehabilitation, and repairs)
23799-New	Other Heavy and Civil Engineering Construction (includes new work; includes projects involving water resources (e.g., dredging and land drainage), development of marine facilities, and projects involving open space improvement (e.g., parks and trails))
23799-Repair	Other Heavy and Civil Engineering Construction (includes reconstruction, rehabilitation, and repairs; includes projects involving water resources (e.g., dredging and land drainage), development of marine facilities, and projects involving open space improvement (e.g., parks and trails))
481	Air freight
482	Rail freight
483	Water freight
484	Truck freight
493	Warehousing and Storage
5321	Automotive Equipment Rental and Leasing
5324	Commercial and Industrial Machinery and Equipment Rental and Leasing
5411	Legal Services
54121	Accounting, Tax Preparation, Bookkeeping, and Payroll Services
5413	Architectural, Engineering, and Related Services (includes landscape architecture, building inspections, surveying and mapping, and testing laboratories)
541511	Custom Computer Programming Services
541512	Computer Systems Design Services
54161	Management Consulting Services
54162	Environmental Consulting Services
54169	Other Scientific and Technical Consulting Services
5417	Scientific Research and Development Services
54182	Public Relations Agencies
54194	Veterinary Services
54199	All Other Professional, Scientific, and Technical Services
56173	Landscaping Services (includes planting trees and shrubs; does NOT include landscape architecture)
562	Waste Management and Remediation Services
81111	Automotive Mechanical and Electrical Repair and Maintenance
81121	Electronic and Precision Equipment Repair and Maintenance
81131	Commercial and Industrial Machinery and Equipment (except Automotive and Electronic) Repair and Maintenance
813	Religious, Grantmaking, Civic, Professional, and Similar Organizations

**Table A3:** North American Industrial Classification System (NAICS) codes relevant to restoration materials purchases. Codes are based on 2017 NAICS (Office of Management and Budget 2017; see https://www.census.gov/naics/)

NAICS code	NAICS description
1114	Nursery/greenhouse products
11194	Mulch/Hay/Straw
111998	Seeds
21231	Mining and quarrying stone
21232	Sand and Gravel
31321	Natural fiber fabrics
31323	Synthetic fabrics
321999	Wood products including wood fencing
32411	Gasoline
325311	Fertilizer and soil amendments
32551	Paint and other coatings
326122	Plastic pipes and pipe fittings
32619	Plastic netting
32732	Ready-mix concrete
32733	Brick/concrete products including concrete pipes and conduits
3311	Iron or steel products, including metal posts
3325	Hardware
3326	Wire fencing
3342	Communications equipment
33611	Vehicle purchase (cars)
336112	Vehicle purchase (light duty trucks)
336120	Vehicle purchase (heavy duty trucks)

**Table A4:** North American Industrial Classification System (NAICS) codes relevant to restoration realty and legal services. Codes are based on 2017 NAICS (Office of Management and Budget 2017; see https://www.census.gov/naics/)

NAICS code	NAICS description
522	Banking, Loans, Brokerage, and Other Loan Intermediary Costs
531	Real Estate Services (realtors, appraisals, property management)
5411	Legal Services

**Restoration spending and analysis details for the hypothetical Pennsylvania stream restoration project**

Tables A5–A7

**Table A5:** Project cost data for a hypothetical Pennsylvania stream restoration project. Project cost data are organized using the Restoration Cost Collection (RCC) framework

#	Activity name	Activity type	Activity cost	Activity dates	Implementing party	Location of expenditure	NAICS code or expenditure breakdown
1	Planning restoration activities	Planning	$125,000	10/2016–09/2017	State/local govt	Lackawanna County, Pennsylvania	Labor hours: 2000 Labor costs: $100,000 Travel costs: $0 Other: $25,000 (overhead)
2	Engineering and design services	Planning	$65,000	01/2017– 06/2017	Private Industry	Dauphin County, Pennsylvania	Architectural, Engineering, and Related Services (5413)
3	Archaeological Surveys	Planning	$25,600	03/2017–05/2017	Private Industry	Baltimore County, Maryland	Architectural, Engineering, and Related Services (5413)
4	Project management and oversight	Project management and/or oversight	$250,000	10/2016–09/2019	State/local govt	Wyoming County, Pennsylvania	Labor hours: 3120 Labor costs: $225,000 Travel costs: $0 Other: $25,000 (overhead and software)
5	Water quality monitoring	Monitoring	$95,000	01/2017–09/2019	State/local govt	Dauphin County, Pennsylvania	Labor hours: 1400 Labor costs: $70,000 Travel costs: $6,000 Other: $19,000 (overhead)
6	Biological Surveys: Benthic macroinvertebrate and drone geomorphology	Monitoring	$72,000	01/2017–09/2019	Private Industry	Onondaga County, New York	Environmental Consulting Services (54162)
7	Biological Surveys: Fish monitoring surveys and data analysis	Monitoring	$33,500	01/2017–09/2017	State/local govt	Centre County, Pennsylvania	Labor hours: 960 Labor costs: $24,000 Travel costs: $4,500 Other: $5,000 (instrumentation)
8	Biological Surveys: Fish monitoring surveys and data analysis	Monitoring	$55,000	01/2019–09/2019	State/local govt	Centre County, Pennsylvania	Labor hours: 1740 Labor costs: $43,500 Travel costs: $6,500 Other: $5,000 (instrumentation)
9	Biological Surveys: Data processing and analysis	Monitoring	$8,400	03/2019–09/2019	Private Industry	Onondaga County, New York	Environmental Consulting Services (54162)
10	Rock and riprap	Materials purchase - direct	$895,000	07/2017–07/2017	Private Industry	Wyoming County, Pennsylvania	Mining and quarrying stone (21231)
11	Rock and riprap	Materials purchase - direct	$925,000	05/2018–05/2018	Private Industry	Wyoming County, Pennsylvania	Mining and quarrying stone (21231)
12	Grass and shrub seeds for embankment	Materials purchase - retail	$65,000	03/2019–03/2019	Private Industry	Lackawanna County, Pennsylvania	Seeds (111998)
13	Tree saplings	Materials purchase - wholesale	$185,000	03/2019–03/2019	State/local govt	Centre County, Pennsylvania	Nursery/greenhouse products (1114)
14	Breach demolition, removal of 2 dams, and installation of fish passage structures	Implementation	$1,875,000	06/2017–09/2018	Private Industry	Dauphin County, Pennsylvania	Other Heavy and Civil Engineering Construction (23799-Repair)
15	Bank stabilization, erosion control, and recontouring	Implementation	$865,000	06/2017–09/2018	Private Industry	Lackawanna County, Pennsylvania	Other Heavy and Civil Engineering Construction (23799-Repair)
16	Dredging for sediment removal	Implementation	$790,000	04/2018–09/2018	Private Industry	Dauphin County, Pennsylvania	Other Heavy and Civil Engineering Construction (23799-New)
17	Electrical work to bury public utility lines	Implementation	$15,000	07/2017–07/2017	Private Industry	Wyoming County, New York	Power and Communication Line and Related Structures Construction (23713-New)
18	Construction of multi-use hard surface trail for recreation	Implementation	$375,000	03/2019–05/2019	Private Industry	Wyoming County, Pennsylvania	Other Heavy and Civil Engineering Construction (23799-New)
19	Fence construction	Implementation	$24,500	06/2019–07/2019	Private Industry	Baltimore County, Maryland	Landscaping Services (56173)
20	Planting grass and trees	Implementation	$51,200	03/2019–05/2019	NGO	Wyoming County, Pennsylvania	Landscaping Services (56173)
21	Interpretative signs and interactive educational materials	Implementation	$75,000	01/2019–07/2019	NGO	Washington County, Vermont	Environmental Consulting Services (54162)
22	Inspection of electrical activities	Implementation	$8,500	08/2017–08/2017	Private Industry	Wyoming County, Pennsylvania	Architectural, Engineering, and Related Services (5413)
23	Inspection of construction activities	Implementation	$15,000	10/2018–10/2018	Private Industry	Wyoming County, Pennsylvania	Architectural, Engineering, and Related Services (5413)
24	Attorney to help secure land purchase	Realty - legal services	$36,000	09/2017–02/2018	Private Industry	Dauphin County, Pennsylvania	Legal Services (5411)
25	Purchase of ten acres of land adjacent to open space	Realty - land purchase	$750,000	02/2018–02/2018	Private Industry	Wyoming County, Pennsylvania	Leaked from impact analysis
	Total Project Expenditures		$7,679,700

**Table A6:** Bridge linking project cost data for the hypothetical Pennsylvania stream restoration project (from Table A5) to the IMPLAN model. Project impacts are estimated using IMPLAN 2019 data (IMPLAN model 2019 data).

#	Restoration activity	Activity dates	Location of expenditure	NAICS code/breakdown sub-category	Total spending ($)	Spending local to PA ($)	IMPLAN event type	IMPLAN 546 sector	IMPLAN dollar year	Additional details
1	Planning restoration activities	10/2016–09/2017	Pennsylvania	Labor Costs	100,000	100,000	Labor Income Change	5001 - Employee Compensation	2016	LPP = 100%
				Travel Costs	-	-	Industry Change	See Travel Breakdown in Table A7	2016	For LPP see Travel Breakdown in Table A7
				Other (overhead)	25,000	25,000	Industry Change	469 - Management of companies and enterprises	2016	LPP = 100%
2	Engineering and design services	01/2017–06/2017	Pennsylvania	5413 - Architectural, Engineering, and Related Services	65,000	65,000	Industry Change	457 - Architectural, engineering, and related services	2016	LPP = 100%
3	Archaeological Surveys	03/2017–05/2017	Maryland	5413 - Architectural, Engineering, and Related Services	25,600	-	Industry Change	457 - Architectural, engineering, and related services	2017	LPP = 100%
4	Project management and oversight	10/2016–09/2019	Pennsylvania	Labor Costs	225,000	225,000	Labor Income Change	5001 - Employee Compensation	2016	
				Travel Costs	-	-	Industry Change	See Travel Breakdown in Table A7	2016	For LPP see Travel Breakdown in Table A7
				Other (overhead)	12,500	12,500	Industry Change	469 - Management of companies and enterprises	2016	LPP = 100%
				Other (software)	12,500	12,500	Commodity Change	3428 - Software Publishers	2016	LPP = SAM, Margins = Producer Price
5	Water quality monitoring	01/2017–09/2019	Pennsylvania	Labor Costs	70,000	70,000	Labor Income Change	5001 - Employee Compensation	2017	LPP = 100%
				Travel Costs	6000	70,000	Industry Change	See Travel Breakdown in Table A7	2017	For LPP see Travel Breakdown in Table A7
				Other (overhead)	19,000	70,000	Industry Change	469 - Management of companies and enterprises	2017	LPP = 100%
6	Biological Surveys: Benthic macroinvertebrate and drone geomorphology	01/2017–09/2019	New York	54162 - Environmental Consulting Services	72,000	-	Industry Change	463 - Environmental and other technical consulting services	2017	LPP = 100%
7	Biological Surveys: Fish monitoring surveys and data analysis	01/2017–09/2017	Pennsylvania	Labor Costs	24,000	24,000	Labor Income Change	5001 - Employee Compensation	2017	LPP = 100%
				Travel Costs	4500	4500	Industry Change	See Travel Breakdown in Table A7	2017	For LPP see Travel Breakdown in Table A7
				Other (instrumentation)	5000	5000	Commodity Change	3312 - Search, detection, and navigation instruments	2017	LPP = SAM, Margins = Purchaser Price
8	Biological Surveys: Fish monitoring surveys and data analysis	01/2019–09/2019	Pennsylvania	Labor Costs	43,500	43,500	Labor Income Change	5001 - Employee Compensation	2019	LPP = 100%
				Travel Costs	6500	6500	Industry Change	See Travel Breakdown in Table A7	2019	For LPP see Travel Breakdown in Table A7
				Other (instrumentation)	5000	5000	Industry Change	312 - Search, detection, and navigation instruments manufacturing	2019	LPP = SAM
9	Biological Surveys: Data processing and analysis	03/2019–09/2019	New York	54162 - Environmental Consulting Services	8400	-	Industry Change	463 - Environmental and other technical consulting services	2019	LPP = 100%
10	Rock and riprap	07/2017–07/2017	Pennsylvania	21231 - Mining and quarrying stone	895,000	895,000	Commodity Change	3028 - Stone	2017	LPP = 100%, Margins = Producer
11	Rock and riprap	05/2018–05/2018	Pennsylvania	21231 - Mining and quarrying stone	925,000	925,000	Commodity Change	3028 - Stone	2018	LPP = 100%, Margins = Producer
12	Grass and shrub seeds for embankment	03/2019–03/2019	Pennsylvania	111998 - Seeds	65,000	65,000	Commodity Change	3010 - All other crops	2019	LPP = SAM, Margins = Purchaser Price
13	Tree saplings	03/2019–03/2019	Pennsylvania	1114 - Nursery/greenhouse products	185,000	185,000	Commodity Change	3006 - Greenhouse, nursery, and floriculture production	2019	LPP = SAM, Margins = Purchaser Price
14	Breach demolition, removal of 2 dams, and installation of fish passage structures	06/2017–09/2018	Pennsylvania	23799 - Other Heavy and Civil Engineering Construction	1,875,000	1,875,000	Industry Change	56 - Construction of other new nonresidential structures	2017	LPP = 100%
15	Bank stabilization, erosion control, and recontouring	06/2017–09/2018	Pennsylvania	23799 - Other Heavy and Civil Engineering Construction	865,000	865,000	Industry Change	56 - Construction of other new nonresidential structures	2017	LPP = 100%
16	Dredging for sediment removal	04/2018–09/2018	Pennsylvania	23799 - Other Heavy and Civil Engineering Construction	790,000	790,000	Industry Change	56 - Construction of other new nonresidential structures	2018	LPP = 100%
17	Electrical work to bury public utility lines	07/2017–07/2017	New York	23713 - Power and Communication Line and Related Structures Construction	15,000	-	Industry Change	52 - Construction of new power and communication structures	2017	LPP = 100%
18	Construction of multi-use hard surface trail for recreation	03/2019–05/2019	Pennsylvania	23799 - Other Heavy and Civil Engineering Construction	375,000	375,000	Industry Change	56 - Construction of other new nonresidential structures	2019	LPP = 100%
19	Fence construction	06/2019–07/2019	Maryland	56173 - Landscaping Services	24,500	-	Industry Change	477 - Landscape and horticultural services	2019	LPP = 100%
20	Planting grass and trees	03/2019–05/2019	Pennsylvania	56173 - Landscaping Services	51,200	51,200	Industry Change	477 - Landscape and horticultural services	2019	LPP = 100%
21	Interpretative signs and interactive educational materials	01/2019–07/2019	Vermont	54162 - Environmental Consulting Services	75,000	-	Industry Change	463 - Environmental and other technical consulting services	2019	LPP = 100%
22	Inspection of electrical activities	08/2017–08/2017	Pennsylvania	5413 - Architectural, Engineering, and Related Services	8500	8500	Industry Change	457 - Architectural, engineering, and related services	2017	LPP = 100%
23	Inspection of construction activities	10/2018–10/2018	Pennsylvania	5413 - Architectural, Engineering, and Related Services	15,000	15,000	Industry Change	457 - Architectural, engineering, and related services	2018	LPP = 100%
24	Attorney to help secure land purchase	09/2017–02/2018	Pennsylvania	5411 - Legal Services	36,000	36,000	Industry Change	455 - Legal services	2017	LPP = 100%
25	Purchase of ten acres of land adjacent to open space	02/2018–02/2018	Pennsylvania		750,000	750,000				Leak from model

Activity spending is matched to North American Industrial Classification System (NAICS) codes, or is broken into labor, travel, and other nonlabor costs. Only spending that happened within the Commonwealth of Pennsylvania (PA) is included in the model (i.e., restoration activities with Location of Expenditure not equal to Pennsylvania are leaked from the impact analysis). Services are modeled as IMPLAN industry change events with the local purchase percent (LPP) set to 100%. Materials purchased direct from the manufacturer are modeled as IMPLAN commodity change events, with LPP set to 100% and margins set to producer price. Retail and wholesale purchases are modeled as IMPLAN commodity change events with LPP set to the region’s Social Accounting Matrix (SAM) Regional Purchase Coefficients (RPCs) and with margins set to purchaser price; LPP is set to SAM for these purchases because, although we know that the retail or wholesale purchase happened within the modeled region, we do not know if the commodity was produced within the region. Labor costs are modeled as IMPLAN labor income change events; labor income change events only model the secondary (ripple effects) of labor spending. To estimate the direct effects of labor we set direct labor income, value added, and economic output equal to the reported labor costs and estimate direct job-years by dividing reported labor hours by 2080 h (the estimated number of labor hours in a full work year). Travel costs are broken into food, transportation, lodging, automobile, and retail expenditures (see Table A7 for travel cost breakdown). The IMPLAN dollar year is set to the starting year for the activity date range

**Table A7:** Example IMPLAN bridge for travel expenditures. The data presented here represent hypothetical travel cost breakdowns; these data can be used for analysis or can be replaced if better data are available. [LPP: Local Purchase Percentage]

Spending group	Allocation to spending group	IMPLAN event type	IMPLAN 546 sector	Allocation within spending group	Final allocation to IMPLAN sector	Additional details
Food services	29%	Industry Change	509 - Full-service restaurants	50%	14.4%	LPP = 100%
		Industry Change	510 - Limited-service restaurants	25%	7.2%	LPP = 100%
		Industry Change	511 - All other food and drinking places	25%	7.2%	LPP = 100%
Public transportation	22%	Industry Change	414 - Air Transportation	80%	8.9%	LPP = 100%, final allocation for air transportation was cut by 50%
		Industry Change	418 - Transit and ground passenger transportation	20%	4.4%	LPP = 100%
Lodging	22%	Industry Change	507 - Hotels and motels, including casino hotels	100%	22.2%	LPP = 100%
Auto transportation	19%	Industry Change	450 - Automotive equipment rental and leasing	85%	16.1%	LPP = 100%
		Industry Change	408 - Retail - Gasoline stores	15%	2.8%	LPP = 100%
Retail	8%	Industry Change	412 - Miscellaneous store retailers	100%	7.8%	LPP = 100%

## Footnotes

1 The 2021 Infrastructure Investment and Jobs Act includes $2.1 billion appropriated to the Department of the Interior (DOI) and the U.S. Department of Agriculture (USDA) to explicitly implement ecosystem restoration (Title VIII, Section 40804). In addition, funds from BIL and the bipartisan Repairing Existing Public Land by Adding Necessary Trees Act (REPLANT Act) are planned to be used to plant 1 billion trees to address an extensive reforestation backlog in the U.S. (U.S. Department of Agriculture 2022). The Executive Order to “Strengthen America’s Forests, Boost Wildfire Resilience, and Combat Global Deforestation,” will leverage funds from BIL to help communities reduce wildfire risk by improving overall forest health (United States, Executive Office of the President 2022b). Additional support from Executive Order 14008, “Tackling the Climate Crisis at Home and Abroad,” directs the U.S. Federal government to support projects that would reclaim abandoned mines and oil and gas wells, creating jobs in economically disadvantaged and rural communities. The $1 billion America the Beautiful Challenge is a public-private partnership leveraging Federal resources with private and philanthropic contributions to support and accelerate locally led conservation and restoration projects (United States, Executive Office of the President 2022a).

2 Economic impacts are different from economic benefits, as defined in cost-benefit analyses. Economic benefits measure the net value or welfare changes to society resulting from ecosystem recovery. Jobs and business activity supported by restoration activities are a complementary, socially relevant outcome that measures the distributional benefits to individual workers and communities that stem from spending on restoration activities. Restoration spending falls into the cost column in cost-benefit analyses. Therefore, economic impact estimates are not appropriate for inclusion in economic cost-benefit analyses and should not be viewed or claimed as benefits by potential responsible parties (see Department of the Interior 2012 for more information on impact analysis compared to cost-benefit analysis, along with examples of benefits from conservation, mitigation, reclamation, and restoration).

3 Jobs are annualized (i.e., 1 job represents 12 months of employment, not necessarily 1 employee). Job-years are used to describe employment for multi-year projects.

4 Labor income includes employee wages and payroll benefits and the incomes of sole proprietors.

5 Value added is equal to the sum of the values added to a product at each step of the production chain, equal to the value of the production of goods and services less the cost of intermediate expenditures. The components of value added are labor income, taxes, profits, and rents.

6 Economic output sums sales along each step of the production chain, this includes the sum of all intermediate sales (business to business sales) and final sales (sales to consumers).

7 https://www.census.gov/naics/

8 https://apps.bea.gov/regional/rims/rimsii/

9 https://implan.com/

10 Any use of trade, firm, or product names is for descriptive purposes only and does not imply endorsement by the U.S. Government.
